# Quality of life, functional outcome, and voice handicap index in partial laryngectomy patients for early glottic cancer

**DOI:** 10.1186/1472-6815-6-1

**Published:** 2006-01-17

**Authors:** Tolga Kandogan, Aylin Sanal

**Affiliations:** 1 Department of Otolaryngology & Head-Neck Surgery, SSK Izmir Hospital, Izmir, Turkey; 2 Selen Ses Merkezi, Ali Cetinkaya Bulvari No:31/1 Daire 24 Alsancak Izmir 35220 Turkey

Following the publication of this paper [1], BioMed Central learned of the publication of a paper by the same authors in another journal [2] using overlapping data on the same set of patients, specifically in regards to the voice handicap index.      

Dr. Kandogan regrets any possible confusion.  

## Pre-publication history

The pre-publication history for this paper can be accessed here:


